# Pancreatic Progenitors and Organoids as a Prerequisite to Model Pancreatic Diseases and Cancer

**DOI:** 10.1155/2019/9301382

**Published:** 2019-02-25

**Authors:** Meike Hohwieler, Martin Müller, Pierre-Olivier Frappart, Sandra Heller

**Affiliations:** Department of Internal Medicine 1, Ulm University Medical Centre, Albert-Einstein-Allee 23, 89081 Ulm, Germany

## Abstract

Embryonic stem cells (ESCs) and induced pluripotent stem cells (iPSCs) are characterized by their unique capacity to stepwise differentiate towards any particular cell type in an adult organism. Pluripotent stem cells provide a beneficial platform to model hereditary diseases and even cancer development. While the incidence of pancreatic diseases such as diabetes and pancreatitis is increasing, the understanding of the underlying pathogenesis of particular diseases remains limited. Only a few recent publications have contributed to the characterization of human pancreatic development in the fetal stage. Hence, most knowledge of pancreatic specification is based on murine embryology. Optimizing and understanding current *in vitro* protocols for pancreatic differentiation of ESCs and iPSCs constitutes a prerequisite to generate functional pancreatic cells for better disease modeling and drug discovery. Moreover, human pancreatic organoids derived from pluripotent stem cells, organ-restricted stem cells, and tumor samples provide a powerful technology to model carcinogenesis and hereditary diseases independent of genetically engineered mouse models. Herein, we summarize recent advances in directed differentiation of pancreatic organoids comprising endocrine cell types. Beyond that, we illustrate up-and-coming applications for organoid-based platforms.

## 1. Introduction

Recent advances in stem cell research have resulted in a multitude of new tools for superior disease modeling of both hereditary diseases and cancer development. The incidence rates of pancreatic diseases such as diabetes and pancreatitis are rising and prognosis of pancreatic cancer is poor [1–3], resulting in a high demand for new technologies that will advance knowledge and improve future therapeutic approaches. While a few recent studies have utilized human fetal pancreas for gene expression studies, the majority of knowledge regarding the complex signaling interplay in pancreatic development is derived from mouse models [4–6]. This reveals the unmet need to optimize *in vitro* differentiation protocols for the development of functional human endocrine and exocrine pancreatic cells essential for disease modeling or drug development [7].

The introduction of induced pluripotent stem cell (iPSC) technology represented a huge step in advanced *in vitro* modeling and disease-specific drug screening for inherited diseases. Takahashi et al. and Takahashi and Yamanaka demonstrated that the enforced expression of OCT4, SOX2, Klf4, and c-Myc in fibroblasts was able to reprogram these cells to a pluripotent stem cell state [8, 9]. These iPSCs exhibit key features of embryonic stem cells isolated from the inner cell mass of the blastocyst, e.g., the expression of transcription factors (OCT4, SOX2, and NANOG) and cell surface markers (SSEA-3 and SSEA-4) [8, 9].

Patient-specific iPSCs as well as embryonic stem cells (ESCs) harbor hallmarks of pluripotency as they are characterized by their limitless ability to self-renew as well as to differentiate into any cell type in the body [10]. Therefore, they may serve as a source for *in vitro* differentiation into different cell types of the pancreatic lineage. Protocols aim to recapitulate embryonic development with stage-specific modulation of particular signaling including Wnt, Notch, Sonic hedgehog (SHH), and bone morphogenetic protein (BMP) leading to the sequential induction of the definitive endoderm (DE), gut tube endoderm (GTE), pancreatic endoderm (PE), and pancreatic progenitor (PP) stages [7, 11–15]. Combined use of small molecules and growth factors efficiently generates multipotent pancreatic progenitors [13–17], subsequently differentiating into ductal, acinar, and endocrine lineages [18]. However, the signaling networks leading to both specification and maturation of all pancreatic cell types are still not fully understood [19].

Organoids represent an important step forward in the functional *in vitro* modeling of the pancreatic tissue. 3D organoid cultures with functional and structural properties of the adult pancreas can be derived from pluripotent stem cells or organ-restricted stem cells [20] and therefore are useful for analyzing basic gene functions and cellular processes. In addition, this technology might be helpful in translational medicine and modeling of hereditary diseases and carcinogenesis as well as in regenerative medicine [20, 21].

This review summarizes recent progress in the establishment of pancreatic lineage derivatives from PSCs and provides an overview of the potential application of organoids as model systems for hereditary pancreatic diseases, diabetes, and pancreatic cancer.

## 2. Main Text

The pancreas is a compound gland with an exocrine compartment comprised of acinar and ductal cells and an endocrine compartment containing alpha, beta, gamma, epsilon, and PP cells which are organized in Langerhans islets [22–24]. Various diseases affect the pancreas arising from defects in different compartments. Diabetes mellitus (DM) represents the most frequent endocrinologic disease accompanied with an increasing prevalence in all industrialized countries [25, 26]. While different subtypes of DM show different facets of extrapancreatic metabolic dysregulation, they all exhibit intrapancreatic *β*-cell dysfunction as a key component. DM type 1 is characterized by a loss of insulin-producing *β*-cells due to autoimmune destruction or dysfunction, whereas type 2 is triggered by insulin resistance and lower insulin secretion. Moreover, other forms of DM can be caused by gene mutations affecting pancreatic development or function. *In vitro* differentiation of PSCs increases our understanding of pancreatic development and disease as underlying mechanisms can be studied chronologically in a highly defined manner.

### 2.1. Regulation of Pancreatic Differentiation

ESCs harbor a complex and tightly regulated signaling network to maintain the proliferative and undifferentiated state *in vivo*. Transcription factors OCT4 and SOX2 as well as NANOG control the expression of pluripotency factors and suppression of lineage-specific genes [27–29]. These intrinsic stem cell regulators are connected with highly interactive signaling pathways facilitating self-renewal in response to extrinsic factors such as FGF or TGF*β* [30, 31]. In order to promote and maintain this pluripotent state artificially *in vitro*, ESCs and iPSCs are cultured on feeder cells such as murine or rat embryonic fibroblasts providing a matrix for cellular attachment and essential signaling cues for survival and proliferation, requiring additional supplementation with basic FGF and serum (or serum replacement) [32, 33]. Alternatively, feeder cells can be replaced by extracellular matrix components and culture medium substituted with multiple growth factors supporting *in vitro* cell growth [34].

For further *in vitro* differentiation, PSCs can recapitulate embryonic development generating pancreatic cells. *In vivo*, pluripotent cells of the inner cell mass form the three primary germ layers of the embryo with endo-, meso-, and ectoderm [35]. The first branch towards pancreatic lineage commitment is the specification into DE originating from the most anterior primitive streak region by modulated Wnt, Nodal, and BMP signaling [36, 37]. *In vitro*, this signaling is mimicked with Wnt3A or GSK3*β* inhibition and TGF*β* ligand Activin A, inducing the expression of typical cellular DE markers SOX17, FOXA2, CXCR4, and c-Kit [11, 38]. After gastrulation, the DE forms the primitive gut tube followed by the anterior-posterior patterning resulting in organ specification, where a dorsal and ventral pancreatic bud is formed at the posterior foregut domain [36, 37]. Besides intrinsic signaling of endodermal cells, the patterning is also mediated by extrinsic signaling from surrounding extracellular matrix and mesodermal cells such as pancreatic mesenchyme, notochord, and dorsal aorta [39]. *In vitro*, these processes are recapitulated with FGF signaling and subsequent treatment with retinoic acid with parallel BMP signal inhibition through Noggin or LDN193189 [11, 12, 15, 38, 40]. Moreover, SHH signaling is blocked with SANT-1 leading to the cellular expression of PDX1, PTF1A, HNF6, and FOXA2, when pancreatic endoderm stage is reached [12, 15, 41]. *In vivo*, the pancreatic buds are composed of multipotent pancreatic progenitor cells that during subsequent morphogenesis give rise to more committed pancreatic cell types. Further expansion of the pancreatic progenitor pool is mediated by FGF10-induced proliferation with expression of PDX1, NKX6.1, SOX9, CPA1, PTF1A, and FOXA2 at the pancreatic progenitor stage [41–43]. Also, Notch signaling plays an important role at multiple stages; however, the precise regulation during pancreatic development in humans is not fully understood. In mice, differential Notch signaling in the early pancreatic endoderm is thought to serve as a gatekeeper between progenitor amplification and differentiation preventing premature differentiation of exocrine and endocrine cells and maintaining pancreatic progenitors [44, 45]. Moreover, Notch signaling seems to be involved in duct specification providing an extensive cue in the determination of different cell types [45].

**Figure 1:**
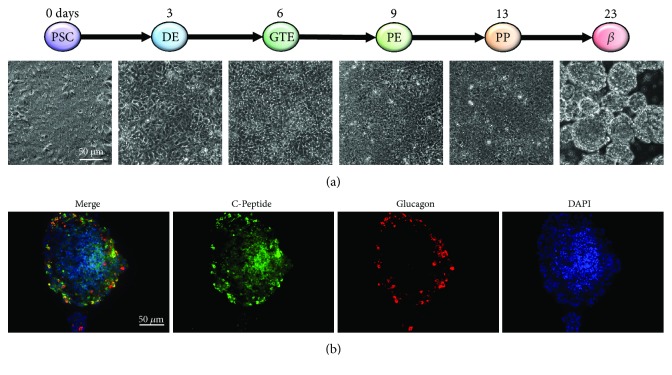
Stages of *in vitro* pancreatic development generating endocrine cells. (a) Stepwise differentiation of the human ESC line HUES8 with representative phase contrast images of the respective pancreatic lineage with 2D culture from the PSC stage until the PP stage, sphere culture at *β*-cell stage. (b) Immunofluorescence staining of cells from *β*-cell stage for C-peptide (*β*-cell marker, green) and glucagon (*α*-cell marker, red).

For analysis of more mature stages *in vitro*, pancreatic progenitor cells can be further differentiated to obtain *β*-cells. Nevertheless, current endocrine differentiation protocols for monolayer/2D cultures are not very efficient and yield only an immature or mixed population of polyhormonal endocrine cells [41]. Moreover, insulin secretion in response to glucose stimulation, a prerequisite of *β*-cells, is absent [40, 41]. However, transplantation of these cells into immunodeficient mice gives rise to mature *β*-cells suggesting a premature *in vitro* phenotype [12, 46].  Figure 1 depicts the different stages of stepwise *in vitro* pancreatic endocrine differentiation with exemplary images of the human ESC line HUES8 (Figure 1(a)). For this cell line, we adapted the differentiation protocol from the Nostro lab [7, 17] resulting in polyhormonal cells (Figure 1(b)).

**Figure 2:**
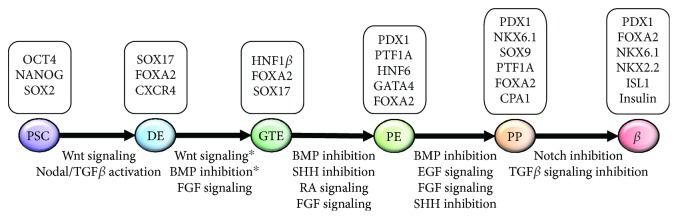
Schematic outline of endocrine lineage commitment *in vitro*. Overview of stepwise and stage-specific modulation of cellular signaling pathways during *in vitro* differentiation summarizing the main protocols. For each pancreatic lineage, specific transcription factors and cellular markers are shown. ^∗^According to the Nostro protocol [17].

**Figure 3:**
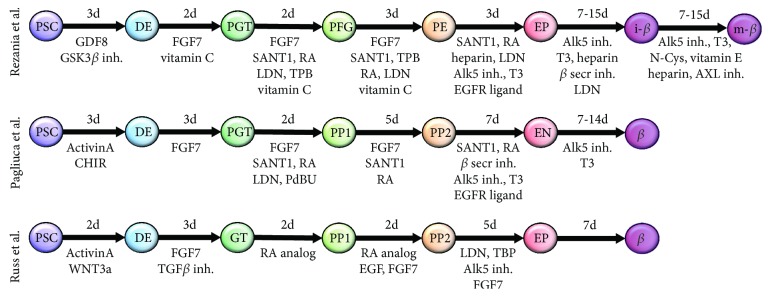
Schematic overview of stepwise pancreatic endocrine differentiation protocols. Stage-specific modulation is conducted by the addition of different growth factors and small molecules. The duration of each stage is depicted in days. PSC: pluripotent stem cells; DE: definitive endoderm; PGT: primitive gut tube; PFG: posterior foregut; PE: pancreatic endoderm; EP: pancreatic endocrine precursors; i-*β*: immature *β*-cells; m-*β*: mature *β*-cells; PP1: PDX1^+^ pancreatic progenitors; PP2: PDX1^+^/NKX6.1^+^ pancreatic progenitors; EN: NKX6.1^+^/C-peptide^+^ cells.

New *in vitro* differentiation protocols including a 3D culture step are promising as they result in glucose-responsive *β*-like cells. The three current and most promising protocols for pancreatic *β*-cell differentiation will be compared (see also Table 1): while the protocols adapted from Pagliuca et al. [13] and Russ et al. [15] describe a suspension culture system with spinner flasks and orbital shaker, respectively, Rezania et al. [14] start differentiation in monolayer cells and changes to an air-liquid interphase culture at the pancreatic endoderm stage. The various stage-specific signaling pathways modulated by the differentiation regimens of these most recent protocols [13–15] are summarized in Figure 2, which also depicts the respective transcription factors and cellular markers expressed during *in vitro* differentiation [5, 47, 48]. Moreover, Figure 3 gives a summary of stage-specific growth factors and small molecules and points at the slightly different nomenclatures of the different stages during pancreatic endocrine differentiation.

Induction of the definitive endoderm and primitive gut tube by modulation of the commonly used Activin and Wnt signaling followed by the FGF family protein FGF7 is employed in all three protocols. In addition, the Kieffer protocol applied vitamin C at early stages (d3-10); SHH and BMP inhibitors, PKC (protein kinase C) activator, and retinoic acid after the primitive gut tube stage and supplements the media with thyroid hormone, EGFR ligand betacellulin, heparin, and Notch inhibitors at later stages for the generation of functional *β*-cells. The differentiation protocol of the Melton lab includes similar growth factors and small molecules as shown in the Kieffer protocol. Additionally, Notch inhibition is used after the PP2 stage. In the Hebrok lab, DE is induced similarly followed by FGF signaling in combination with the inhibition of TGF*β* signaling. Furthermore, after reaching the gut tube stage, retinoic acid is added for only three days followed by the stimulation of EGF signaling and FGF signaling until cells become pancreatic progenitors. This stage is followed by the inhibition of BMP signaling, the activation of PKC, and the continued FGF signaling for differentiation to endocrine progenitors. The final maturation to functional *β*-cells is carried out in the media without any additional growth factors.

For all three protocols, the optimization of differentiation conditions leading to the generation of a high number of PDX1^+^/NKX6.1^+^ double-positive pancreatic progenitors seems necessary for the increased endocrine differentiation leading to more functional *β*-cells. Whereas Hebrok provides a short protocol of 3 weeks, Kieffer and Melton differentiate the ESCs for 4-5 weeks to induce functional *β*-like cells. Overall, all protocols lead to a high percentage of NKX6.1^+^/PDX1^+^ cells, and at least 25% of cells are double positive for PDX1 and C-peptide or insulin. Moreover, *β*-like cells secreted insulin after glucose challenge *in vitro*, and after transplantation into mice, human C-peptide was detected within 2 weeks supporting a more mature stage of *β*-like cells than observed in 2D differentiation culture.

These new pancreatic differentiation protocols show that 3D culture conditions promote maturation and improve the function of *in vitro*-generated *β*-cells. *In vitro* pancreatic differentiation of PSCs provides a promising alternative to diabetes treatment with cadaveric islets in regenerative medicine. For instance, the use of pancreatic endoderm cells derived from an hESC line as a type 1 diabetes therapy is currently being tested in a clinical trial in the USA (NCT02239354) [49]. Although *in vitro* pancreas differentiation seems promising to model pancreatic diseases, limitations need to be overcome for use in regenerative medicine. So far, current protocols are lacking standardization for multiple cell lines and only result in premature *β*-cells. Moreover, scaling up the process as well as maintaining *β*-cells in a culture would be useful to meet the need in therapy. However, the novel technology of encapsulating insulin-producing cells in microdiscs combined with *in vitro*-generated *β*-cells will advance the treatment of type 1 diabetic patients [50].

### 2.2. PSCs to Model Pancreatic Inherited Diseases and Diabetes

Better protocols not only provide tools to generate *β*-like cells but also contribute a valuable platform to model pancreatic diseases and development and to characterize the stage-specific role of certain genes. Additionally, genome editing by CRISPR/Cas9-mediated knockout, correction of a gene of interest [51, 52], or the generation of disease-specific iPSCs [9] combined with the organoid technology are accompanied by many potential applications [53, 54]. Based on the previously mentioned differentiation protocol, *β*-cells were generated from iPSCs of type 1 diabetes (T1D) patients to study functionality as well as their response to antidiabetic drugs [55]. Although T1D *β*-cell function is similar to nondiabetic *β*-cells, the developed stress model might prove useful for drug screening and as a discovery platform. Recent work from Shi et al. used CRISPR/Cas9-edited hPSCs to investigate multigenic human traits associated with neonatal and adult-onset diabetes [56]. Loss of one GATA6 allele was shown to affect pancreatic differentiation and formation of glucose-responsive *β*-cells. Moreover, this model revealed a complex genetic interaction with GATA4 expanding the application to characterize genetic modifiers in diseases such as T2D and to identify novel therapeutic targets. Similarly, iPSCs from a pancreatic agenesis patient harboring a GATA6 mutation were differentiated to investigate the pancreatic developmental defect revealing that GATA6 plays an important role during endoderm formation and function of *β*-cells [57]. McGrath et al. investigated the role of neurogenin3 (NGN3) in human pancreatic endocrine development [58]. In mice, NGN3 is essential for the development of endocrine cell types; however, patients with NGN3 mutations reported to be amorphic still develop a functional pancreas. The complete knockout of NGN3 in hESCs resulted in differentiation towards pancreatic progenitor cells but not endocrine cells. In hESCs with siRNA-mediated knockdown, the remaining NGN3 was sufficient for the generation of endocrine cells suggesting that mutations in patients lead to decreased NGN3 protein activity and development of functional endocrine pancreas. Moreover, developing novel approaches to differentiate hPSCs towards *β*-cells in microfluidic devices will help to provide automated and high-throughput systems for drug screening and patient-specific therapy [59].

For more functional analyses, organoids from PSCs allow the study of biological processes and tissue-specific responses. Recently, iPSCs from cystic fibrosis (CF) patients were used to characterize the impact of cystic fibrosis transmembrane conductance regulator (CFTR) on pancreatic commitment and to model pancreatic aspects of the disease [54]. Induced PSCs from CF patients showed unaltered pancreatic development; however, CF pancreatic organoids mirrored the defective CFTR function typical for these patients. As a proof of concept, these CF organoids were used to screen for CFTR correctors and activators providing a novel platform for PSC-based drug screening and testing of therapeutic procedures.

### 2.3. Pancreatic Ductal Adenocarcinoma

Pancreatic ductal adenocarcinoma (PDAC) represents one of the most lethal malignancies [60]. Pancreatic precursor lesions such as pancreatic intraepithelial neoplasia (PanIN) promoted by chronic pancreatitis give rise to PDAC development [61]. Both high mortality and increasing incidence of PDAC require more efficient therapies and a better understanding of pathobiology. However, suitable *ex vivo* models of the pancreas are missing. Since transformed cell lines cannot recapitulate the complexity of a native organ and developmental, genetic, and physiological differences in animal models exhibit their limitations [53, 62], PSCs provide a valuable tool for disease modeling further elucidating the cellular and molecular mechanisms underlying the disease.

### 2.4. Tumor-Derived Organoids in PDAC Research

Pioneering work in establishing intestinal organoid systems was carried out by the group of Hirsch et al. and Yui et al. [63, 64]. After an initial determination of an intestinal stem cell marker (Lgr5), the group transferred this finding to the pancreas: under physiological conditions, Lgr5 is not expressed in the pancreas, but under conditions caused by mechanical damage, Lgr5 positive cells appear as a cradle of regeneration effort. Vice versa, Huch et al. were able to demonstrate that Wnt-agonistic R-spondins do not only induce Lgr5 expression in cell cultures from primary murine pancreatic tissue but also contribute to maintain pancreatic organoid growth from adult murine ductal cells in a 3D matrix [65, 66].

For PDAC, various model systems, based either on distinct tumor cell line genetically engineered mouse models (GEMMs) or on xenograft models, have been established over the last years. Research on all these platforms increased our understanding of basic genetics of tumorigenesis, tumor progression, and formation of metastases. However, all of them imply relevant limitations regarding the extent of accuracy for patient-specific disease modeling and therapeutic drug screening. Any prior tumor models fail to mimic tumor heterogeneity but also lack the tumor- and patient-specific microenvironment that represents a major impediment for therapeutic response. Thus, model systems of lower complexity are not able to predict therapeutic responses for individual patients, whereas more sophisticated systems like patient-derived xenografts need up to 12 months until a sufficient maturation is reached [67–69]. For PDAC, a relevant proportion of patients will not survive until this individual therapeutic screening platform can be established. On the other hand, GEMMs are able to imitate key features of human PDAC, especially tumor-initiating complexes like PanIN lesions [70]. Although GEMMs led to an amplification of our understanding of the key features of PDAC and disease-specific characteristics in drug delivery, this approach of disease modeling does not incorporate aspects of personalized medicine.

**Figure 4:**
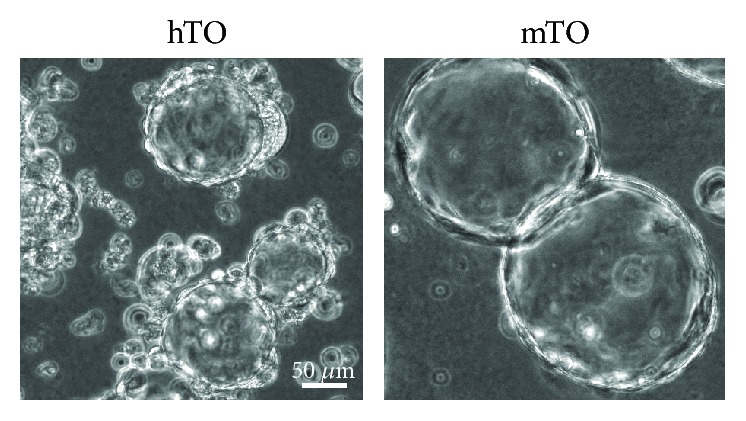
Pancreatic organoids in a 3D matrigel culture. Phase contrast images of human tumor-derived organoids (hTO) generated from PDAC samples and mouse tumor-derived organoids (mTO) generated from pancreatic tumors in the KC (KRAS^G12D^, p48-Cre) mouse model.

To overcome these limitations, organoids from human PDAC tumor tissue were successfully generated. Organoids can be derived from mature, healthy tissue types such as the intestine, prostate, liver, and pancreas [65, 66, 71, 72] as well as from transformed cancer tissue [73–75]. A key feature in organoid cultures is their defined growth under circumvention of attachment on unphysiological surfaces resulting in a 3-dimensional culture that comprises stem cell-driven self-renewal and self-organization. Organoids can even contain different cell types, thus presenting a complex organ structure [76]. Organoids derived from mature primary tissues do not contain connective tissue types (stromal or mesenchymal cell types). Therefore, this deficit has to be compensated with an extracellular basement membrane-like matrix containing laminin, collagen, and proteoglycans. In contrast to organoids derived from healthy tissue, those generated from PDAC samples (human tumor-derived organoids, hTOs, see Figure 4) mostly resemble PanIN lesions under 3D culture conditions even at the time of organoid initiation [75]. In order to generate pancreas 3D organoids, tumor or healthy tissue is disintegrated mechanically and/or with collagenase followed by embedding in a collagen or matrigel matrix. For sustained growth, organoids have to be supplemented with growth factors and modulators such as epidermal growth factor, fibroblast growth factor 10, Noggin, Wnt3A, gastrin, nicotinamide, N-acetylcysteine, R-spondin, and an Alk inhibitor [75]. Other protocols include insulin, hydrocortisone, ascorbic acid, retinoic acid, fibroblast growth factor 2, and Rock inhibitor in the organoid culture medium [20].

Targeted DNA sequencing revealed critical mutations in tumor-associated genes like *KRAS*, *TP53*, *SMAD*, *CKDN2A*, and others within the hTOs but not in organoids from healthy patients (hNOs) [75]. Once transplanted into nude (*Nu/Nu*) mice, human tumor-derived organoids will form infiltrative carcinoma within months, inducing a strong desmoplastic reaction with a stroma-rich microenvironment [77]. Moreover, key genes that are upregulated in progressive PDAC can also be detected in these tumors, emphasizing the capacity of disease modeling with hTOs. In order to predict clinical behavior, PDAC was classified into the classical and quasi-mesenchymal (QM) subtype based on gene expression data of tumor samples [78]. Since hTOs reach a high neoplastic purity in culture [79], transcriptomic and functional profiling enables refinement of classification. Seino et al. identified three new functional subtypes according to dependency of hTOs on stem cell niche factors Wnt and R-spondin that can help to predict clinical behavior or treatment options [80]. Also, while the classical subtype is not well represented in PDAC cell line models, hTOs allow culture of both subtypes [78]. Additionally, Tiriac et al. identified two specific molecular subtypes that correlate with the classical and QM subtype but describe unique gene expression programs underlining the benefit of hTOs in identification of PDAC subtype signatures.

Interestingly, only pancreatic duct cells but not endocrine or acinar cells are able to form organoids [65, 75]. As in hNOs, hTOs exclusively express markers of ductal cells, but not of other pancreatic cell lines. Moreover, the regeneration of *β*-cells during islet neogenesis occurs by self-duplication of remaining *β*-cells [81] or *α*-cell conversion [82], suggesting that pancreatic stem cells reside in ductal tissue. This is also reflected by the proliferation potential of Doublecortin-like kinase-1 (Dclk1)^+^ cells found in the mouse pancreas. These quiescent cells reside mainly in the ductal epithelium in a healthy pancreas and can contribute to tissue regeneration during injury and inflammation. Moreover, Dclk1^+^ cells form organoids with continued organoid growth and might play a role as cancer stem cell or tumor-initiating cell [83]. In adult human pancreatic tissue, a subpopulation with high aldehyde dehydrogenase activity shows the potential for organoid expansion with a more limited capability of regeneration compared to organoids derived from murine pancreatic tissue [84]. Better understanding of specific factors and signaling for maintaining the stem cell population in culture will help to generate long-lived organoids from human tissue.

Tumor-derived organoids maintain a given tumor phenotype with a continuing ability to progress into locally invasive and metastatic carcinomas upon orthotopic transplantation [75]. Currently, the value of hTOs in predicting a patient-specific response for a distinct therapeutic regime remains to be determined; however, current work on other gastrointestinal cancer types is promising [85]. Recent publications have already implemented hTOs as a screening platform for the evaluation of new therapeutic strategies in pancreatic cancer: Kumar et al. were able to show a limited organoid growth of hTOs after chemical inhibition of MAPK-interacting protein kinases by inducing a reversal of EMT (epithelial-mesenchymal transition) [86]. Seino et al. pointed out the loss of stem cell niche factor (Wnt) dependence in organoids derived from progressive tumor subtypes, allowing a novel insight into the niche/stromal cancer crosstalk [80].

For subsequent clinical application, hTOs can be generated from small patient samples taken from a fine needle aspiration that has to be obtained in most of the patients within the routine diagnostic of pancreatic cancer [87]. Organoid models of higher complexity involve the coculture of tumor organoids with stromal and immune cell components of the patient in order to mimic a tumor microenvironment [88]. This model partially addresses the interaction of cancer-associated fibroblasts and immune cell infiltrates with the tumor. Therefore, it might provide a more accurate patient-derived organoid model for the testing of drug responses and allow evaluation of the effect of immunotherapeutics in PDAC.

Despite the promising hopes provided by organoid culture, several limitations remain: First, most of the studies, especially in the pancreas field, fail to integrate both the immune and stromal components while these factors are key modulators of drug response and drug scavenging [88]. Any incorporation of those components in clinical high-throughput screening still represents a technical challenge. Second, the clinical prediction of *in vivo* drug concentrations based on *in vitro* drug concentrations used in organoid culture remains imprecise: performing IC50 measurements for each drug and each patient is definitely unrealistic in a clinical setting mainly due to limited cell availability and time restrictions caused by the need for early clinical decision-making. Additionally, reference curves determining whether a certain IC50 will predict a patient response are not yet available. It is surprising that despite 10 years of organoids' “rebirth” story and their use in preclinical studies, the following basic, technical, and mechanistic questions have not been answered or standardized: What is the appropriate size of organoids that are most representative of the tumor response to drugs? How long do organoids have to be exposed to drugs to mimic the drug-tumor response? Finally, one of the central questions that needs to be addressed is the evolution of the tumor itself and its acquisition of resistance during treatment. Current settings of organoid drug screenings only allow analysis of organoids derived from a previous disease state. Indeed, in a clinical view, the patient will always receive the first line chemotherapy (as an example in PDAC either FOLFORINOX or GEMCITABINE+PACTITAXEL) before the organoid culture will reach the point to be used for drug screening. Therefore, possible drug-induced resistance mechanisms will remain inaccessible for organoid screens from a single biopsy. A novel approach to address these limitations was lately published by Tiriac et al.: a systematic analysis of organoid-based gene expression signatures was correlated with a clinical response to different therapeutic regimes [79]. Moreover, organoids resistant to clinical chemotherapeutics show improved response for investigative and targeted agents providing alternative treatment options in a reasonable timeframe. Such a combined approach enables a more precise PDAC subtype classification. Also, a sufficient correlation between organoids and patient responses could be improved. Repetitive tumor biopsies in a single patient may more adequately factor tumor evolution within the course of the disease, and combined therapeutic, genomic, and transcriptomic organoid-based profiling might enhance current strategies of personalized medicine. Another study using liquid biopsy showed that some mutations arise rapidly in response to treatment [89]. Few additional approaches have been suggested to overcome this problem: First, the use of a “minimal” medium will select the most aggressive and niche factor-independent tumor organoids [80]. Second, the establishment of organoids might be performed directly from circulating tumor cells (CTCs) [90, 91], even if the isolation of CTCs remains challenging.

Taken together, the latest data indicate that organoids represent one of the most promising tools as significant progress was made in predicting patients' drug response and better delineation of underlying mechanisms of tumor progression. The opportunity of a patient-specific screening platform for individualized therapeutic strategies with a potential to reevaluate therapeutic options at later stages of tumor progression represents an essential advantage of this approach associated with limited invasiveness. However, a lot of procedural improvements are required to reach the optimum use in a clinical approach.

## 3. Conclusion

With regard to iPSC/ESC-based organoid technology, significant progress was achieved in the recent past. The fast-evolving technical advancements in pancreatic organoid biology was realized via an intricate optimization of pancreatic differentiation protocols. In doing so, *in vitro* differentiation protocols of human ESCs have been adapted by knowledge obtained from mouse development to generate various functional pancreatic cell types within an organoid culture. However, even though optimized use of different small molecules and growth factors as well as ESC suspension culture improved *β*-cell differentiation, protocols are found to be rather exclusive for specific cell lines.

The establishment of tumor-derived organoids shows great promise for new approaches in both dissection of tumorigenesis and patient-specific optimization of therapeutic options. But still, the simulation of specific tumor-(micro)environment interaction represents a pending effort in the field.

**Figure 5:**
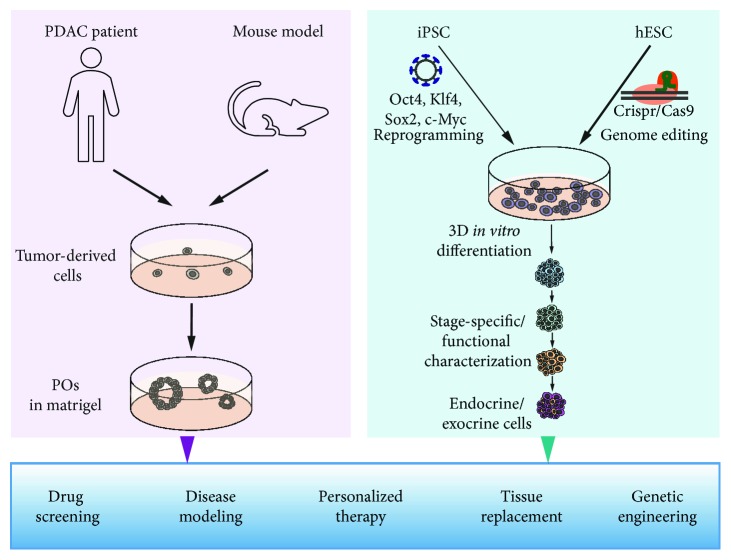
Pancreatic organoids and PSCs to improve therapy and understanding of pancreatic diseases.

In summary, organoid systems have already proven to promote pancreatic research leading to progress especially within the field of diabetes and PDAC (Figure 5).

## Figures and Tables

**Table 1:** Comparison of *in vitro* pancreatic endocrine differentiation protocols.

	Kieffer (Rezania et al.)	Melton (Pagliuca et al.)	Hebrok (Russ et al.)
Culture system	Monolayer followed by air-liquid interphase	Suspension in spinner flasks	Suspension on orbital shaker
Cell lines	H1, iPSC line	HUES8, 2 iPSC lines	Mel1 Ins^GFP/W^
Divergent conditions	Vitamin C from PGT stage to PE stage; protein kinase C (PKC) activator TPB after the PGT and PFG stages; SHH/BMP inhibition after the PGT stage; thyroid hormone, heparin, and EGFR ligand after the PE stage; vitamin E analog, AXL inhibitor, and N-Cys at the *β*-cell stage; total 27-43 days	Media change every other day after d2; PKC activator PdBU after the PGT stage; Notch inhibition and EGF signaling after the PP2 stage; total 27-34 days	TGF*β*-inhibitor after the DE stage; RA analog only in high glucose medium after the GT stage; EGF after the PP1 stage; BMP inhibition and FGF signaling after the PP2 stage; no growth factors after the EP stage; shortened time intervals (total 21 days)
Efficiency PP	70% NKX6.1^+^/PDX1^+^ (PP), 76% (immature *β*-cells)	>55% NKX6.1^+^/PDX1^+^ (PP2)	80% NKX6.1^+^/PDX1^+^ (d9)
Efficiency beta cells	40% PDX1^+^/INS^+^ (maturing *β*-cells)	33% PDX1^+^/C-peptide^+^ (functional *β*-cells)	25% PDX1^+^/C-peptide^+^ (*β*-like cells)
*In vivo* transplantation (mouse)	Human C-peptide within 2 weeks after transplantation, ameliorates hyperglycemia after d40 posttransplantation	Human C-peptide within 2 weeks after transplantation, ameliorates progressive hyperglycemia	Human C-peptide within 7-10d after transplantation, reduces STZ-induced hyperglycemia
